# Open questions: a few that need answers in immunology

**DOI:** 10.1186/1741-7007-11-115

**Published:** 2013-11-26

**Authors:** Brigitta Stockinger

**Affiliations:** 1 Division of Molecular Immunology, MRC National Institute for Medical Research, Mill Hill, London NW7 1AA, UK

## 

The cells of the immune system fall into two broad categories - innate and adaptive - with the cells of the innate immune system providing frontline defense by means of invariant receptors to conserved microbial components, and the B and T lymphocytes of the adaptive immune system, with highly variable somatically diversified receptors, providing reinforcement after selective proliferation in response to specific antigen. While this principle still holds, the ‘old’ view of these systems as developmentally separate and functionally distinct has been eroded, most recently and dramatically by the discovery of a whole new group of innate lymphoid cell subsets. Another development has been the increasing emphasis on studying immune responses *in vivo* in interaction with stromal components that are beginning to be seen as essential contributors to the development of immune responses, rather than just as matrix or scaffold components in lymphoid organs or non-lymphoid peripheral tissues. A major focus now is on immune defense at epithelial barriers and homeostatic coexistence with a healthy microbiota, as it is becoming increasingly clear that intestinal dysbiosis strongly influences immune responses even at distal sites. However, whether dysbiosis is a cause or consequence of dysregulated inflammatory responses remains to be unraveled.

I outline below some of the open questions raised by these developments.

## Innate lymphoid cell biology

Innate lymphoid cells (ILCs) have dominated the recent literature and by now many of their developmental and functional properties as well as distinct lineages have been identified [1,2]. These cells, unlike T lymphocytes, do not have antigen-specific receptors, but they do secrete many of the cytokines whereby classical adaptive T lymphocytes execute their functions in activating other cells. It is remarkable how many features of the adaptive immune system are replicated by ILCs and it is tempting to speculate that they arose during evolution as part of a defense system that preceded adaptive immunity. Nevertheless it remains unclear what precise impact these cells have, as it is not yet possible to selectively deplete just ILC subsets.

The relative resilience of severely immunocompromised mouse strains under standard laboratory conditions suggests that ILCs and other innate immune cells may compensate to some degree for an absence of adaptive immune cells. Whether the development of an adaptive immune response requires prior activation and collaboration with the corresponding ILC population is not clear.

## Functions of IL-17-producing cell types

A subset of T helper cells known as Th17 cells and other IL-17-producing T cells have been in the limelight for a few years now, initially because they were implicated in autoimmune and inflammatory pathology, although it is now also apparent that they have a role in immune homeostasis in the intestine, and that together with other IL-17-producing cells they are important for immune defense against a range of fungi and bacteria [3]. Despite considerable insights into their role *in vivo*, however, our knowledge of many of their features is still based on deductions from cells grown in *in vitro* culture systems.

It appears that such cells may not necessarily replicate the full functional profile of *in vivo* generated cells and in any case most investigation has focused on TCRαβ-expressing Th17 cells and less is known about specific contributions of IL-17-producing TCRγδ subsets (found systemically, and in the lung, reproductive tract, oral cavity and skin) or IL-17-producing natural killer T subsets or the enigmatic thymic TCRαβ IL-17 poised T-cell population. It is conceivable that they all have distinct functional contributions, which need to be dissected in infection models taking advantage of cytokine reporter constructs to track the fate of activated effector cells. It also remains to be elucidated what the relationship is between intestinal Th17 cells that maintain barrier homeostasis in steady state and IL-23 driven pathogenic Th17 cells that cause autoimmune or hyperinflammatory disorders. Furthermore, the specific contributions of IL-17- and or IL-22-secreting cells of the ILC3 subset in the intestine remain to be elucidated.

## Nuclear receptors and their crosstalk with the immune system

Several reports have linked nuclear receptors such as LXR, RAR, RXR, VDR, and AhR to immune system functions, although the underlying mechanisms remain undefined in most cases. These receptors provide tantalizing links between metabolic pathways and immune parameters, such as inflammatory responses and autoimmunity [4,5]. Signals received through such receptors influence the responses of both innate and adaptive immune cells. A dissection of nuclear receptor crosstalk as well as their specific and combined influence on the immune system in steady state and inflammation will undoubtedly yield important insights into regulatory pathways that shape the function of participating cells.
